# Determinants of Left Ventricular Systolic Function One Year after Primary Percutaneous Coronary Intervention for ST-elevation Myocardial Infarction in a Middle-Income Country

**DOI:** 10.34172/aim.2023.15

**Published:** 2023-02-01

**Authors:** Reza Heidari Moghadam, Nahid Salehi, Susan Mahmoudi, Lida Shojaei, Sirus Nasiri, Soraya Siabani, Parisa Janjani, Mohammad Rouzbahani, Hooman Tadbiri, Mahdi Nalini

**Affiliations:** ^1^Cardiovascular Research Center, Health Institute, Kermanshah University of Medical Sciences, Imam Ali Hospital, Kermanshah, Iran; ^2^Department of Health Education and Health Promotion, Kermanshah University of Medical Sciences, Kermanshah, Iran

**Keywords:** Cardiovascular disease, Ejection fraction, Ischemic heart disease

## Abstract

**Background:**

Little is known about the predictors of left ventricular ejection fraction (LVEF) —an important predictor of mortality— after primary percutaneous coronary intervention (PCI) in low- and middle-income countries.

**Methods:**

In a prospective cohort study at Imam Ali hospital, Kermanshah, Iran, we enrolled consecutive ST-elevation myocardial infarction (STEMI) patients treated with primary PCI (2016-2018) and followed them up to one year. LVEF levels were measured by echocardiography, at baseline and one-year follow-up. Determinants of preserved/improved LVEF were assessed using multi-variable logistic regression models.

**Results:**

Of 803 patients (mean age 58.53±11.7 years, 20.5% women), baseline LVEF levels of ≤35% were reported in 44%, 35- 50% in 40%, and ≥50% in 16% of patients. The mean ± SD of LVEF increased from 38.13%±9.2% at baseline to 41.49%±9.5% at follow-up. LVEF was preserved/improved in 629 (78.3%) patients. Adjusted ORs (95% CIs) for predictors of preserved/improved LVEF showed positive associations with creatinine clearance, 1.01 (1.00-1.02) and adherence to clopidogrel, 2.01 (1.33-3.02); and inverse associations with history of myocardial infarction (MI), 0.44 (0.25-0.78); creatine kinase MB (CK-MB), 0.997 (0.996- 0.999); door-balloon time (3^rd^ vs. 1^st^ tertile), 0.62 (0.39-0.98); number of diseased vessels (2 and 3 vs. 1: 0.63 (0.41-0.99) and 0.58 (0.36-0.93), respectively); and baseline LVEF (35-50% and ≥50% vs. ≤35%: 0.45 (0.28-0.71) and 0.19 (0.11-0.34), respectively).

**Conclusion:**

Adherence to clopidogrel, short door-balloon time, high creatinine clearance, and lower baseline LVEF were associated with preserved/improved LVEF, while history of MI, high CK-MB, and multi-vessel disease were predictors of reduced LVEF. Long-term drug adherence should be considered for LVEF improvement in low- and middle-income countries.

## Introduction

 Primary percutaneous coronary intervention (PCI) has dramatically improved the prognosis of patients with acute ST-elevation myocardial infarction (STEMI).^1-3^ Nonetheless, there is considerable variability in survival rate after primary PCI, and outcomes remain suboptimal, especially in low- and middle-income contraries (LMICs).^4^

 Left ventricular ejection fraction (LVEF) is one of the most powerful predictors of short-term and long-term mortality and morbidity following myocardial infarction (MI).^5-8^ Primary PCI can restore coronary patency to re-perfuse the myocardial tissue and thus preserve left ventricular function.^1^ During the months after initial treatment, LVEF may improve by mechanisms such as remodeling and gradual relief of myocardial stunning.^9,10^

 Identifying the determinants of LVEF changes may have significant implications for both prognostic and therapeutic objectives.^11^ Although some studies in high-income countries have shown that clinical, laboratory, angiographic, and pharmaceutical variables can predict LVEF changes,^12-18^ data on long-term LVEF changes after primary PCI in LMICs are scarce. We therefore sought to evaluate the predictors of one-year LVEF changes after primary PCI for STEMI in a tertiary care heart hospital in western Iran.

## Materials and Methods

###  Study Design, Setting, and Participants

 In this prospective cohort study, we enrolled consecutive adult patients (>18 years) with STEMI who underwent primary PCI between July 1, 2016 and October 30, 2018, in Imam Ali hospital and followed them up to one year. This university hospital is the only hospital with primary PCI facility in the Kermanshah province, with a population of almost 2 million, in western Iran. All patients with successful primary PCI and one-year follow-up were included in our study. Exclusion criteria were unwillingness to participate and unsuccessful revascularization by primary PCI, defined as coronary artery bypass graft surgery (CABGs) before hospital discharge.

###  Baseline Assessment

 Data on demographic variables, past medical history, cardiac risk factors, signs and symptoms, and laboratory tests including serum creatinine and the MB isoenzyme of creatine kinase (CK-MB) activity levels were recorded. Baseline creatinine clearance was calculated based on the Modification of Diet in Renal Disease (MDRD) equation.^19^ PCI characteristics including date and time, the number and type of diseased vessel, the epicardial thrombolysis in myocardial infarction (TIMI) flow^20^ before and after the procedure, and stent insertion were obtained. We determined the time from hospital admission to balloon inflation (door-balloon time). All patients underwent 2-dimensional echocardiographic examinations within 24-48 hours after the index infarction using a commercially available machine (vivid3) with 2.5- and 3.5-MHz transducers. A standard imaging protocol was used based on apical 4- and 2-chamber views. All measurements of LVEF were performed by board certified echo-cardiologists, blinded to the current study. Echocardiogram reports were reviewed and the LVEF was recorded. Medications at discharge, including aspirin, adenosine diphosphate inhibitors, angiotensin-converting enzyme inhibitors, angiotensin receptor blockers, beta-blockers, and statins were also recorded.

###  One-Year Follow-up

 One year after the primary PCI, all patients were invited to the hospital. Trained nurse interviewers, using a structured questionnaire, collected detailed data on health status, medications, and admissions to hospitals. LVEF was re-evaluated using the same echocardiography protocol, free of charge.

###  Statistical Analysis

 Preserved or improved LVEF, one year after primary PCI, was the main outcome of this study. Participants were categorized into two groups based on the LVEF changes from baseline to follow-up: patients with reduced LVEF (LVEF at 1-year follow-up less than baseline LVEF) and patients with preserved or improved LVEF. Logistic regression models were used to determine the predictors of persisting/improved LVEF. Both crude and adjusted odds ratios (ORs) with 95% confidence intervals (CIs) were reported. Predictor variables were selected based on the previous studies and variables of interest, including baseline LVEF, age, sex, body-mass index (kg/m^2^), ever smoking tobacco, diabetes, dyslipidemia, hypertension, past history of PCI, MI, and CABGs, clearance of creatinine (mL/min), peak CK-MB activity (U/L), percutaneous arterial access, number of diseased vessels, left anterior descending artery PCI, TIMI flow before and after PCI, stent insertion, door-balloon time (minutes, tertiles), non-culprit lesion treatment, discharge and follow-up medications, and follow-up events (i.e. acute coronary syndrome, PCI, and CABGs). Independent predictors of preserved LVEF were determined by multivariable logistic regression analysis using stepwise selection of variables with entry and exit criteria of a *P* value < 0.15.^18^ We conducted three sensitivity analyses: (1) after exclusion of patients with heart events during follow-up (n=182); (2) after exclusion of patients with baseline LVEF ≥ 50% (n=130); and (3) we defined decreased LVEF as a reduction of at least 10% in LVEF,^21^ considering an error margin of classification, and re-evaluated our results. In a subgroup analysis, we categorized patients based on the median baseline LVEF (i.e. 40%) and evaluated the outcome in the subgroups. Continuous data with normal distributions are expressed as means (SD) and categorical data as numbers (proportion). Continuous data with non-normal distributions are expressed as medians (25^th^, 75^th^ percentiles). *P* values<0.05 or 95% CIs not including one were considered as statistically significant. The Stata statistical software, version 12 (StataCorp, College Station, TX) was used for analyses.

## Results

 Of 1058 consecutive STEMI patients treated with primary PCI, 58 (5.48%) were excluded for CABGs following unsuccessful revascularization by PCI, 26 (2.46%) for death before discharge, 39 (3.69%) for death during follow-up, 94 (8.88%) for unwillingness to participate in the follow-up protocol, and 38 (3.59%) for loss to follow-up. Finally, 803 patients were included in our study.

 There were no missing values for baseline and follow-up LVEF, and for other covariates, the numbers of missing values were small (history of dyslipidemia: 37 (4.61%), previous MI: 25 (3.11%), and TIMI flow after primary PCI: 2 (0.25%)). In these cases, we used separate missing indicators to keep them in the models.

 The mean (SD) age was 58.53 (11.7) years and 20.5% were women. Fifty-one percent of participants had a history of ever smoking tobacco, 18% diabetes, 36% hypertension, and 23% dyslipidemia. Previous MI was reported in 72 (9%), CABGs in 18 (2%) and PCI in 46 (6%) of participants. Thirty percent of patients had 3 diseased vessels. The left anterior descending artery was the infarct vessel in 56% of patients. The median (25^th^, 75^th^ percentiles) of door-balloon time was 95 (78, 127) minutes (Table 1).

**Table 1:** Baseline and One-year Follow-up Characteristics after Primary PCI, Stratified by LVEF Change

**Clinical Characteristics**	**All (n=803)**	**Reduced LVEF (n=174)**	**Preserved/Improved LVEF (n=629)**
Age (y)	58.53 (11.7)	59.94 (12.22)	58.14 (11.55)
Sex, women	164 (20.42)	31 (17.82)	133 (21.14)
Body mass index (kg/m^2^)	26.60 (3.89)	26.22 (3.74)	26.70 (3.93)
Ever smoker	409 (50.93)	94 (54.02)	315 (50.08)
History of diabetes	146 (18.18)	26 (14.94)	120 (19.08)
History of hypertension	290 (36.11)	61 (35.06)	229 (36.41)
History of dyslipidemia^*^	176 (22.98)	40 (24.10)	136 (22.67)
History of myocardial infarction^*^	72 (9.25)	26 (15.29)	46 (7.57)
History of CABGs	18 (2.24)	7 (4.02)	11 (1.75)
History of PCI	46 (5.73)	10 (5.75)	36 (5.72)
Clearance of creatinine (mL/min)	76.06 (60.81, 92.45)	72.98 (59.07, 90.01)	77.84 (61.75, 93.60)
**Procedural Characteristics and Outcomes**			
Highest CK-MB (U/L)	120 (61, 218)	169.5 (75,270)	110 (59, 208)
Percutaneous arterial access			
Femoral	425 (52.93)	96 (55.17)	329 (52.31)
Radial	378 (47.07)	78 (44.83)	300 (47.69)
Number of diseased vessels			
1	268 (33.73)	46 (26.44)	222 (35.29)
2	298 (37.11)	70 (40.23)	228 (36.25)
3	237 (29.51)	58 (33.33)	179 (28.46)
Infarct vessels			
Left anterior descending	448 (55.79)	92 (52.87)	356 (56.60)
Right	265 (33.00)	64 (36.78)	201 (31.96)
Left circumference	83 (10.34)	14 (8.05)	69 (10.97)
Left main	1 (0.12)	1 (0.57)	0.00
Vein graft	6 (0.75)	3 (1.72)	3 (0.48)
TIMI grade 0/1flow before primary PCI	745 (92.78)	164 (94.25)	581 (92.37)
Stent insertion	747 (93.03)	156 (89.66)	591 (93.96)
Door-Balloon time (min)	95 (78,127)	102 (83,149)	95 (77, 124)
TIMI grade 3 flow after primary PCI^*^	768 (95.88)	163 (93.68)	605 (96.49)
Non-culprit lesion treatment	39 (4.86)	9 (5.17)	30 (4.77)
LVEF at discharge			
≤35%	353 (43.96)	59 (33.91)	294 (46.74)
>35%-<50%	320 (39.85)	70 (40.23)	250 (39.75)
≥50%	130 (16.19)	45 (25.86)	85 (13.51)
Discharge medications			
Aspirin	795 (99.00)	173 (99.43)	622 (98.89)
Clopidogrel	798 (99.38)	173 (99.43)	625 (99.36)
ACE-I/ARB	577 (71.86)	129 (74.14)	448 (71.22)
Beta-Blockers	653 (81.32)	138 (79.31)	515 (81.88)
Statin	784 (97.63)	170 (97.70)	614 (97.62)
**One-Year Follow-up**			
PCI	125 (15.57)	32 (18.39)	93 (14.79)
CABGs	36 (4.48)	18 (10.34)	18 (2.86)
Acute coronary syndrome	30 (3.74)	8 (4.60)	22 (3.50)
Medications			
Aspirin	715 (89.04)	152 (87.36)	563 (89.51)
Clopidogrel	630 (78.46)	122 (70.11)	508 (80.76)
ACE-I/ARB	407 (50.68)	81 (46.55)	326 (51.83)
Beta-Blockers	533 (66.38)	110 (63.22)	423 (67.25)
Statin	641 (79.83)	132 (75.86)	509 (80.92)

PCI, percutaneous coronary intervention; LVEF, left ventricular ejection fraction; CABGs, coronary artery bypass graft surgery; CK-MB, creatine kinase MB; TIMI, thrombolysis in myocardial infarction; ACE-I/ARB, angiotensin converting enzyme inhibitors or angiotensin receptor blockers. Data are mean (SD), median (25th, 75th), or n (%). *Missing values: history of dyslipidemia: 37, previous MI: 25, and TIMI flow after primary PCI: 2 patients.

 Mean (SD) LVEF increased from 38.13% (9.2) at baseline to 41.49% (9.5) at one year. Baseline LVEF levels of ≤ 35% were reported in 353 (44%), 35-50% in 320 (40%), and ≥50% in 130 (16%) patients. At the follow-up, these levels were reported in 226 (28%), 339 (42%), and 238 (30%), respectively. An improvement in LVEF was observed in 455 (56.7%) patients, whereas, in 174 patients (21.7%) there was a decrease in LVEF. In the remaining patients (21.7%), no change was observed. The median (25^th^, 75^th^ percentiles) LVEF change (%) was 12.50 (0, 28.57) in patients with preserved or improved LVEF, and 12.50 (9.09, 25.00) in patients with reduced LVEF.

Table 1shows the clinical, procedural, and follow-up characteristics of participants according to the change in LVEF. Although at discharge, almost all patients were advised to use aspirin, clopidogrel, and statin, at one-year follow-up, these drugs were used in 89%, 78%, and 80% of patients, respectively (Table 1). The only adenosine diphosphate inhibitor drug used in the patients was clopidogrel.

Table 2 shows the predictors of preserved or improved LVEF after one-year follow-up, based on crude analyses. History of MI, high levels of CK-MB, low levels of creatinine clearance, more than one coronary vessel disease, long door-balloon time, higher levels of baseline LVEF, and discontinuing clopidogrel intake were associated with reduced LVEF (*P* value for all < 0.05).

**Table 2:** Predictors of Preserved/improved LVEF One Year after Primary PCI: Crude Analyses

**Clinical Characteristics**	**ORs (95% CIs)**	***P*** ** values**
Age	0.99 (0.97-1.00)	0.074
Sex (reference: men)	1.24 (0.80-1.91)	0.336
Body mass index (kg/m^2^)	1.03 (0.99-1.08)	0.149
Ever Smoker	0.85 (0.61-1.20)	0.357
History of diabetes	1.21 (0.81-1.80)	0.351
History of hypertension	1.06 (0.75-1.51)	0.743
History of dyslipidemia	0.96 (0.71-1.29)	0.777
History of myocardial infarction	0.45 (0.27-0.76)	0.003
History of CABGs	0.42 (0.16-1.11)	0.081
History of PCI	1.00 (0.48-2.05)	0.990
Creatinine clearance (mL/min)	1.01 (1.00-1.01)	0.036
**Procedural Characteristics and Outcomes**		
Highest CK-MB(U/L)	0.999 (0.998-1.00)	0.027
Percutaneous arterial access (reference: femoral)	1.12 (0.80-1.57)	0.503
Number of diseased vessels (reference: 1)		
2 vessels	0.67 (0.45-1.02)	0.064
3 vessels	0.64 (0.41-0.99)	0.044
Infarct vessel (LAD vs others)	1.16 (0.83-1.63)	0.382
TIMI grade before primary PCI (0/1 vs 2/3)	0.74 (0.37-1.49)	0.397
Stent insertion	1.79 (1.00-3.23)	0.051
Door-Balloon time (reference: 1^st^ tertile)	
2^nd^ tertile	0.64 (0.42-0.98)	0.040
3^rd^ tertile	0.60 (0.39-0.92)	0.020
TIMI grade after primary PCI (reference: <3)	1.86 (0.88-3.91)	0.103
Non-culprit lesion treatment	0.92 (0.43-1.97)	0.827
LVEF at discharge (reference: ≤35%)		
>35-50%	0.72 (0.49-1.05)	0.090
≥50%	0.38 (0.24-0.60)	<0.001
**Medications at follow-up**		
Aspirin	1.23 (0.74-2.07)	0.422
Clopidogrel	1.79 (1.22-2.62)	0.003
ACE-I/ARB	1.24 (0.88-1.73)	0.218
Beta-Blockers	1.19 (0.84-1.70)	0.319
Statin	1.35 (0.90-2.01)	0.142

LVEF, left ventricular ejection fraction; PCI, percutaneous coronary intervention; CABGs, coronary artery bypass graft surgery; CK-MB, creatine kinase MB; LAD, left anterior descending; TIMI, thrombolysis in myocardial infarction; ACE-I/ARB, angiotensin converting enzyme inhibitors or angiotensin receptor blockers.

 Independent LVEF predictors, using multi-variable analyses, are shown in Table 3.History of MI, high levels of CK-MB, multi-coronary disease, long door-balloon time, and high levels of baseline LVEF remained significant predictors of reduced LVEF. High clearance of creatinine and clopidogrel intake had positive associations with preserved/improved LVEF.

**Table 3:** Independent Predictors^*^ of Preserved/Improved LVEF after Primary PCI at One-Year Follow-up

**Characteristics**	**OR (95% CI)**	***P*** ** values**
Sex (reference: men)	1.50 (0.93-2.41)	0.096
History of myocardial infarction	0.44 (0.25-0.78)	0.005
Clearance of creatinine	1.01 (1.00-1.02)	0.010
Highest CK-MB	0.997 (0.996-0.999)	<0.001
Door-Balloon time (reference: 1^st^ tertile)		
2^nd^ tertile	0.67 (0.43-1.05)	0.079
3^rd^ tertile	0.62 (0.39-0.98)	0.040
LAD culprit	0.68 (0.45-1.03)	0.070
Number of diseased vessels (reference: 1)		
2 vessels	0.63 (0.41-0.99)	0.046
3 vessels	0.58 (0.36-0.93)	0.024
LVEF at discharge (reference: ≤35%)		
>35-50%	0.45 (0.28-0.71)	0.001
≥50%	0.19 (0.11-0.34)	<0.001
Clopidogrel at the follow-up	2.01 (1.33-3.02)	0.001

LVEF, left ventricular ejection fraction; PCI, percutaneous coronary intervention; OR (95% CI), odds ratio (95% confidence interval); CK-MB, creatine kinase MB; LAD, left anterior descending. *Using a multi-variable logistic regression model adjusted for all variables in the table.

Table 4 shows the results of sensitivity analyses. After excluding patients with heart events during the follow-up, weakened associations with LVEF were observed for the number of diseased vessels, door-balloon time, previous MI, and adherence to clopidogrel. All the associations remained significant after excluding patients with LVEF ≥ 50% at baseline. In another sensitivity analysis, using 10% error margin to define LVEF reduction, LVEF decreased more than 10% in 124 patients and the independent predictors of preserved/improved LVEF, with ORs (95% CIs), were clearance of creatinine, 1.01 (1.00-1.02) and clopidogrel use at the follow-up, 1.91 (1.20-3.02) with positive associations; and MI history, 0.38 (0.21-0.67); CK-MB, 0.997 (0.996-0.999); number of diseased vessels (2 vs. 1, 0.59 (0.36-0.98) and 3 vs. 1, 0.57 (0.33-0.97); and LVEF at discharge (>35-50% vs. ≤35%, 0.53 (0.34-0.85) and ≥50% vs. ≤35%, 0.40 (0.22-0.72); with inverse associations. In this sensitivity analysis, using statin at follow-up was also associated with preserved/improved LVEF with OR (95% CI) of 1.67 (1.04-2.66).

**Table 4:** Sensitivity Analyses of the Independent Predictors^*^ of Preserved/Improved LVEF after Primary PCI at One-Year Follow-up

**Characteristics**	**Exclusion of Patients with** **Heart Events** ^**^ ** During Follow-up** **(n=621)**		**Exclusion of Patients with** **Baseline LVEF ≥50%** **(n=673)**	
	**ORs (95% CIs)**	***P*** ** Values**	**ORs (95% CIs)**	***P*** ** Values**
Sex (reference: men)	1.89 (1.05-3.39)	0.033	1.59 (0.92-2.75)	0.094
History of myocardial infarction	0.52 (0.27-1.00)	0.052	0.47 (0.25-0.89)	0.020
Clearance of creatinine	1.01 (1.00-1.02)	0.007	1.01 (1.00-1.02)	0.003
Highest CK-MB	0.998 (0.997-1.00)	0.014	0.997 (0.995-0.998)	<0.001
Door-Balloon time (reference: 1^st^ tertile)				
2^nd^ tertile	0.74 (0.43-1.26)	0.268	0.61 (0.36-1.04)	0.068
3^rd^ tertile	0.64 (0.37-1.08)	0.096	0.51 (0.30-0.86)	0.011
LAD culprit	0.66 (0.40-1.09)	0.107	0.68 (0.42-1.10)	0.113
Number of diseased vessels (reference: 1)				
2 vessels	0.69 (0.43-1.13)	0.144	0.56 (0.34-0.94)	0.029
3 vessels	0.92 (0.52-1.63)	0.769	0.56 (0.32-0.98)	0.042
LVEF at discharge (reference: ≤35%)				
>35-50%	0.49 (0.28-0.83)	0.009	0.43 (0.26-0.69)	0.001
≥50%	0.21 (0.11-0.41)	<0.001	-	-
Clopidogrel at the follow-up	1.59 (0.98-2.57)	0.058	2.00 (1.26-3.18)	0.003

LVEF denotes left ventricular ejection fraction; PCI, percutaneous coronary intervention; CK-MB, creatine kinase MB; LAD, left anterior descending. *Using multi-variable Logistic regression models adjusted for all variables in the table. **Heart event includes acute coronary syndrome, coronary bypass surgery, and PCI.

 Adjusted OR (95% CIs) in subgroup analysis indicated that in patients with the baseline LVEF < 40%, sex (women vs. men: 2.56 (1.07-6.13)) and using beta-blockers at the follow-up (1.89 (1.01-3.51)) were positively associated; and MI history (0.35 (0.16-0.76)); CK-MB (0.998 (0.996-1.00)); and door-balloon time, (2^nd^ vs. 1^st^ tertile: 0.43 (0.19-0.96) and 3^rd^ vs. 1^st^ tertile: 0.33 (0.15-0.74)) were inversely associated with preserved/improved LVEF. Furthermore, in patients with LVEF ≥ 40%, clearance of creatinine (1.01 (1.00- 1.02)) and clopidogrel use at follow-up (2.28 (1.38-3.77)) were positively associated, and CK-MB (0.997 (0.995-0.999)) was inversely associated with preserved/improved LVEF (Supplementary file 1, Tables S1 and S2).

## Discussion

 The present study included a large prospective cohort of patients who were treated with primary PCI for STEMI with one-year follow-up. To the best of our knowledge, this is the first study that evaluates the association of adherence to cardio-protective drugs at follow-up with LVEF changes in LMICs. We showed that the independent determinants of preserved/improved LVEF were drug adherence, especially to clopidogrel therapy, short door-balloon time, high creatinine clearance, and a lower baseline LVEF; while, the independent predictors of reduced LVEF were history of MI, high CK-MB, and multi-vessel disease.

 Adherence to dual anti-platelet therapy, typically aspirin and an adenosine diphosphate inhibitor such as clopidogrel, for a minimum of 12 months after an event, is the cornerstone of STEMI management.^22^ We showed that using clopidogrel at follow-up was independently and significantly associated with preserved/improved LVEF. After exclusion of patients with heart events during the follow-up, this association persisted, although non-significantly. Beyond the platelet-inhibition effects, other cardio-protective effects of adenosine diphosphate inhibitors through some pleiotropic mechanisms are well-described.^23^ Some studies have investigated the effects of drug use at hospital discharge on LVEF changes, without paying enough attention to long-term drug adherence.^13-15,18^ Such analyses can obscure the long-term effects of drug adherence on LVEF changes after primary PCI.

 Unfortunately, long-term drug adherence to cardio-protective drugs, including anti-platelet drugs, is suboptimal, especially in LMICs,^24^ such as Iran.^25^ Overall, the rate of permanent discontinuation of anti-platelet drugs after an acute coronary syndrome is almost 25%.^22^ In our study, although aspirin and clopidogrel were prescribed for almost all patients at discharge, adherence to these drugs, after one year of follow-up, decreased to 89% and 78%, respectively. In a large international study, Yusuf et al^24^ reported that drug adherence is affected by economic factors more than individual factors such as age, sex, education, and history of traditional risk factors. Patients in low-income countries had the lowest rates of drug adherence.^24^ In our country, clopidogrel is several times more expensive than aspirin; thus, the cost of clopidogrel has probably been one of the main reasons for non-adherence in our patients. In a randomized trial involving 301 US hospitals and more than 10 000 patients with MI, a co-payment intervention using vouchers increased the persistent use of adenosine diphosphate inhibitors by 3.3% after one year of follow-up.^26^ Other reasons such as unacceptable side effects, complexity of treatments, and drug interruption for non-cardiac procedures may be also related to lack of appropriate anti-platelet drug adherence.^22,27^ Adherence to the other types of cardio-protective drugs in our study was also associated, although non-significantly, with the better outcome.

 As expected, our findings, in line with other studies,^12,13,16,18^ indicated that history of MI, long time to treatment, and high CK-MB were predictors of reduced LVEF at the follow-up. Our results confirmed the belief that “time is muscle” in the setting of primary PCI and that longer time delays were associated with LV dysfunction.^28^ High levels of CK-MB, in our study, were inversely associated with LVEF improvement. Cardiac biomarkers, such as CK-MB, are typically used as surrogate measures for infarct size, which is one of the best predictors of LVEF after MI.^16^

 We found a significant direct association between renal function and LVEF improvement. Some,^17^ but not all,^13,18^ studies indicated that renal insufficiency was an independent predictor of LVEF at follow-up after STEMI. Chronic kidney disease is a major risk factor for coronary artery disease.^29^ As renal function declines, the prevalence of coronary disease, arteriosclerosis, microvascular disease, left ventricular hypertrophy, and myocardial fibrosis increases.^29^

 Our results clearly showed that multi-vessel disease was inversely associated with LVEF recovery after primary PCI. Some studies have reported similar results.^14,15^ These patients have more extensive coronary disease and may have limited collateral blood flow. Therefore, more severe hibernation due to profound myocardial ischemia may occur.^14^

 In the present study, as reported by others,^13,14,18^ low LVEF at the baseline was independently correlated with preserved/improved LVEF after one-year follow-up. Primary PCI not only can limit infarct size, but can also preserve viable tissue in the infarct area. This potentially viable tissue (stunned myocardium) can be depressed for a long period of time, despite successful primary PCI.^14^ The gradual improvement of this stunned myocardium may be greatest in those with low LVEF at the baseline.^13,14^ We acknowledge that some deaths in patients with the lowest LVEF and without improvement in LVEF might occur before the second LVEF measurement, which may also contribute to this finding that the lowest baseline LVEF is a determinant of LVEF recovery. Also, this finding may be in part a reflection of regression toward the mean.^13,28^ Regression toward the mean is a statistical phenomenon, due to solely random error, that occurs when large or small measurements tend to be followed by less extreme measurements, closer to the mean.^30^ However, in a sensitivity analysis, after excluding patients with baseline LVEF of 50% or more, our results remained unchanged. We also evaluated the results in two subgroups, based on the baseline LVEF, and achieved mainly similar results.

 In contrast with previous studies demonstrating that MI location,^14^ LAD/non-LAD lesion,^15^ and TIMI flow after angioplasty^13,18^ were predictors of LVEF changes, we could not find such associations in our analyses. These factors might be collinear with other variables that were found to be related to LVEF changes in our multi-variable analyses such as CK-MB levels and number of diseased vessels. In our study, TIMI grade 3 flow after primary PCI was reported in almost 96% of patients, indicating complete restoration of epicardial coronary blood flow. However, some studies indicated that in about half of the patients, despite successful restoration of these vessels by PCI, the perfusion of the distal coronary microvasculature was not fully restored, which could be related with increased morbidity and mortality.^31^

 This study has several limitations. First, patients who died before one-year follow-up may have had severe LV dysfunction, which may affect our results about predictors of LVEF changes. Second, LVEF evaluation in this study was based on visual assessment (not quantitative methods) of echocardiography; however, expert cardiologists, blinded to our study design, evaluated LVEF levels. Third, we had no data about some variables which may have provided useful insight into the determinants of LVEF changes including wall motion abnormality, Killip class, and some angiographic characteristic such as the length of lesions, bifurcation lesions, and tortuosity of the infarct vessels.

 In conclusion, after one-year follow-up of primary PCI patients, we showed that the independent determinants of preserved/improved LVEF were adherence to clopidogrel use, short door-balloon time, high creatinine clearance, and a lower baseline LVEF, while the independent predictors of reduced LVEF were history of MI, high CK-MB, and multi-vessel disease.

 Our results may provide valuable therapeutic and prognostic information for physicians to better manage patients treated with primary PCI, especially in LMICs. Our findings highlight that long-term cardio-protective drug adherence must be considered in patients treated with primary PCI. Systematic structured secondary prevention programs using better education of patients, family members, nurses, and physicians about the benefits, safety, and long-term need for essential cardiac drugs; the reduction in medication costs and co-payment approaches for selected expensive drugs; and combination therapies to reduce the numbers and doses of daily drug regimen (i.e. polypills) are needed to improve the long-term use of effective drugs, especially in LMICs. Also, all efforts should be made to shorten time to treatment to improve the long-term LVEF after primary PCI.

## Supplementary Files

Supplementary file 1 contains Tables S1 and S2.
