# Supplementary material for: Determinants of Left Ventricular Systolic Function One Year after Primary Percutaneous Coronary Intervention for ST-elevation Myocardial Infarction in a Middle-Income Country
Source: Arch Iran Med. 2023 Feb 1;26(2):92–9. doi: 10.34172/aim.2023.15 (PMC10685896; doi:10.34172/aim.2023.15)
Supplement: Supplementary file 1 — contains Tables S1 and S2. [file aim-26-92-s001.pdf]

Arch Iran Med. February 2023;26(2):92-99

10.34172/aim.2023.15

Supplementary file 1

Table S1. Subgroup Analysis: Predictors of Preserved/improved LVEF One Year after Primary PCI based on the Baseline LVEF.

**Clinical Characteristics**	LVEF <40%		LVEF ≥40%	
	Full-adjusted* ORs (95% CIs)	P values	Full-adjusted* ORs (95% CIs)	P values
Age	0.99 (0.95-1.03)	0.505	1.00 (0.97-1.03)	0.947
Sex (reference: men)	1.76 (0.63-4.89)	0.280	1.35 (0.66-2.76)	0.417
Body mass index (kg/m^2)	1.01 (0.90-1.13)	0.890	0.98 (0.90-1.06)	0.556
Ever Smoker	0.64 (0.30-1.37)	0.249	1.17 (0.68-2.01)	0.562
History of diabetes	1.49 (0.56-3.96)	0.426	1.57 (0.80-3.10)	0.189
History of hypertension	1.51 (0.71-3.22)	0.281	0.89 (0.52-1.53)	0.677
History of dyslipidemia	1.04 (0.41-2.64)	0.937	0.68 (0.38-1.20)	0.185
History of myocardial infarction	0.29 (0.08-1.04)	0.057	0.47 (0.18-1.25)	0.132
History of CABGs	0.51 (0.06-4.59)	0.544	0.94 (0.20-4.37)	0.939
History of PCI	0.98 (0.19-4.99)	0.981	2.92 (0.76-11.27)	0.120
Creatinine clearance (mL/min)	1.01 (0.98-1.03)	0.626	1.01 (1.00-1.03)	0.102
**Procedural Characteristics and outcomes**				
Highest CK-MB(U/L)	0.996 (0.993-0.998)	<0.001	0.997 (0.995-0.999)	0.008
Percutaneous arterial access (reference: femoral)	1.13 (0.57-2.22)	0.731	1.07 (0.66-1.72)	0.786
Number of diseased vessels (reference: 1)				
2 vessels	0.52 (0.23-1.20)	0.126	0.69 (0.39-1.22)	0.203
3 vessels	0.52 (0.21-1.32)	0.169	0.64 (0.35-1.19)	0.163
Infarct vessel (LAD vs others)	0.49 (0.17-1.43)	0.192	0.66 (0.40-1.09)	0.108
TIMI grade before primary PCI (0/1 vs 2/3)	0.41 (0.07-2.30)	0.310	0.89 (0.36-2.20)	0.800
Stent insertion	2.41 (0.73-8.00)	0.150	1.54 (0.61-3.92)	0.362
Door-Balloon time (reference: 1sttertile)				
2ndtertile	0.38 (0.16-0.90)	0.029	0.77 (0.43-1.37)	0.370
3rdtertile	0.25 (0.10-0.60)	0.002	0.90 (0.50-1.61)	0.723
TIMI grade after primary PCI (reference: <3)	0.57 (0.14-2.39)	0.445	1.70 (0.48-6.02)	0.411
Non-culprit lesion treatment	0.56 (0.14-2.26)	0.411	1.13 (0.38-3.36)	0.820
**Medications at follow-up**				
Aspirin	1.85 (0.67-5.15)	0.238	0.64 (0.23-1.75)	0.380
Clopidogrel	1.34 (0.57-3.14)	0.501	2.43 (1.38-4.30)	0.002
ACE-I/ARB	0.55 (0.26-1.18)	0.125	1.50 (0.90-2.51)	0.119
Beta-Blockers	1.71 (0.79-3.68)	0.173	1.05 (0.63-1.76)	0.853
Statin	1.40 (0.60-3.29)	0.438	0.92 (0.46-1.86)	0.820

LVEF denotes left ventricular ejection fraction; PCI, percutaneous coronary intervention; CABGs, coronary artery bypass graft surgery; CK-MB, creatine kinase MB; LAD, left anterior descending; TIMI, thrombolysis in myocardial infarction; ACE-I/ARB, angiotensin converting enzyme inhibitors or angiotensin receptor blockers.
*Adjusted for all the variables in the table.

Table S2. Subgroup Analysis: the Independent Predictors* of Preserved/improved LVEF after Primary PCI at One-year Follow-up based on the Baseline LVEF.

Characteristics	LVEF <40%		LVEF ≥40%	
	OR (95% CI)	P values	OR (95% CI)	P values
Sex (reference: men)	2.56 (1.07-6.13)	0.035	—	—
History of myocardial infarction	0.35 (0.16-0.76)	0.008	—	—
Clearance of creatinine	1.01 (1.00-1.02)	0.135	1.01 (1.00-1.02)	0.019
Highest CK-MB	0.998 (0.996-1.00)	0.011	0.997 (0.995-0.999)	0.002
Door-Balloon time (reference: 1sttertile)				
2ndtertile	0.43 (0.19-0.96)	0.040	—	—
3rdtertile	0.33 (0.15-0.74)	0.007	—	—
Number of diseased vessels (reference: 1)				
2 vessels	0.48 (0.22-1.04)	0.063	0.73 (0.43-1.26)	0.261
3 vessels	0.53 (0.23-1.22)	0.138	0.65 (0.37-1.15)	0.139
Clopidogrel at the follow-up	—	—	2.28 (1.38-3.77)	0.001
Beta-Blockers at the follow-up	1.89 (1.01-3.51)	0.045	—	—

LVEF denotes left ventricular ejection fraction; PCI, percutaneous coronary intervention; OR (95% CI), odds ratio (95% confidence interval); CK-MB, creatine kinase MB; LAD, left anterior descending.

*Independent predictors were determined by multi-variable logistic regression analysis using stepwise selection of variables with entry and exit criteria of a p value<0.15.[18]
